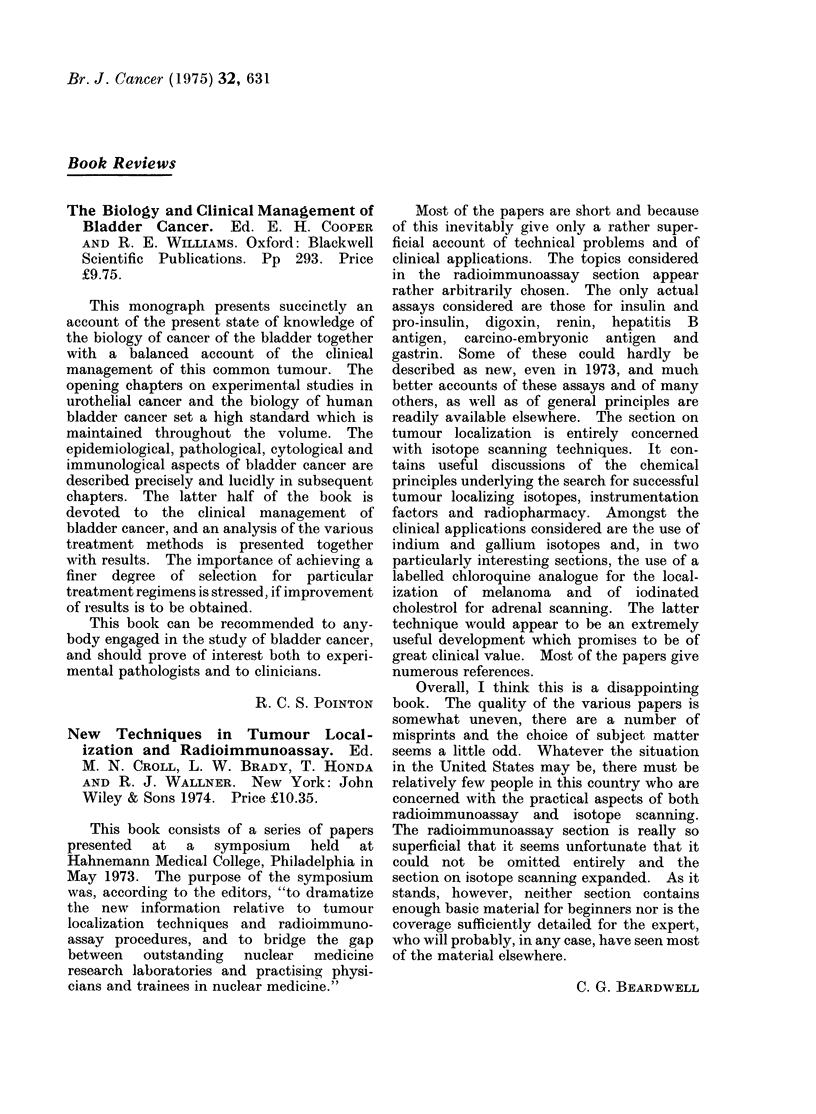# New Techniques in Tumour Localization and Radioimmunoassay

**Published:** 1975-11

**Authors:** C. G. Beardwell


**New Techniques in Tumour Localization and Radioimmunoassay.** Ed. M. N. Croll, L. W. Brady, T. Honda and R. J. Wallner. New York: John Wiley & Sons 1974. Price £10.35.

This book consists of a series of papers presented at a symposium held at Hahnemann Medical College, Philadelphia in May 1973. The purpose of the symposium was, according to the editors, "to dramatize the new information relative to tumour localization techniques and radioimmunoassay procedures, and to bridge the gap between outstanding nuclear medicine research laboratories and practising physicians and trainees in nuclear medicine."

Most of the papers are short and because of this inevitably give only a rather superficial account of technical problems and of clinical applications. The topics considered in the radioimmunoassay section appear rather arbitrarily chosen. The only actual assays considered are those for insulin and pro-insulin, digoxin, renin, hepatitis B antigen, carcino-embryonic antigen and gastrin. Some of these could hardly be described as new, even in 1973, and much better accounts of these assays and of many others, as well as of general principles are readily available elsewhere. The section on tumour localization is entirely concerned with isotope scanning techniques. It contains useful discussions of the chemical principles underlying the search for successful tumour localizing isotopes, instrumentation factors and radiopharmacy. Amongst the clinical applications considered are the use of indium and gallium isotopes and, in two particularly interesting sections, the use of a labelled chloroquine analogue for the localization of melanoma and of iodinated cholestrol for adrenal scanning. The latter technique would appear to be an extremely useful development which promises to be of great clinical value. Most of the papers give numerous references.

Overall, I think this is a disappointing book. The quality of the various papers is somewhat uneven, there are a number of misprints and the choice of subject matter seems a little odd. Whatever the situation in the United States may be, there must be relatively few people in this country who are concerned with the practical aspects of both radioimmunoassay and isotope scanning. The radioimmunoassay section is really so superficial that it seems unfortunate that it could not be omitted entirely and the section on isotope scanning expanded. As it stands, however, neither section contains enough basic material for beginners nor is the coverage sufficiently detailed for the expert, who will probably, in any case, have seen most of the material elsewhere.

C. G. Beardwell